# Case report: Dynamic ^18^F-FDG PET/CT display of a bronchial mass as a second primary cancer mimicking mediastinal lymph node in a gastric carcinoma survivor

**DOI:** 10.3389/fonc.2024.1447843

**Published:** 2024-09-26

**Authors:** Shengyun Huang, Yarong Zhang, Xieraili Wumener, Yuanyuan Lei, Ying Liang

**Affiliations:** ^1^ Department of Nuclear Medicine, National Cancer Center/National Clinical Research Center for Cancer/Cancer Hospital & Shenzhen Hospital, Chinese Academy of Medical Sciences and Peking Union Medical College, Shenzhen, China; ^2^ Department of Pathology, National Cancer Center/National Clinical Research Center for Cancer/Cancer Hospital & Shenzhen Hospital, Chinese Academy of Medical Sciences and Peking Union Medical College, Shenzhen, China

**Keywords:** bronchial mass, dynamic PET/CT, FDG, mucoepidermoid carcinoma, second primary cancer

## Abstract

A 70-year-old woman underwent distal gastrectomy due to gastric adenocarcinoma in 2015. After 6 years, the follow-up CT revealed a suspicious mass in the right hilar of the lung mimicking mediastinal lymph nodes. Further dynamic PET/CT images showed a mass located in the right intermediate bronchus with increased FDG uptake and relatively high Ki value, which may imply the possibility of malignancy. However, the symmetrical mediastinal lymph nodes had intense FDG uptake but relatively low Ki value, suggesting benign lesions. The initial pathological result of the bronchoscopy biopsy was considered suspicious for metastatic gastric adenocarcinoma. However, it was then found consistent with middle-grade mucoepidermoid carcinoma, considered a second primary cancer without metastatic lymph nodes as confirmed by a surgical procedure (lower bilobectomy + hilar and mediastinal lymphadenectomy). ^18^F-FDG PET/CT has an important value in the follow-up of indeterminate findings for patients with a tumor history. Moreover, dynamic quantification parameters such as Ki may be additionally helpful in identifying malignancies in some equivocal situations.

## Introduction

In recent decades, advances in cancer treatment and the aging of the population have led to more frequent survivors of cancer treatment. However, cancer survivors have at least 20% increased risk for secondary cancer compared to the general population (1). In patients with a prior cancer diagnosis, follow-up examinations are performed to rule out relapse and metastasis often over a period of several years. ^18^F-FDG PET/CT has an important role in diagnosis, staging, and restaging of a patient with a malignant tumor. In a large series of 1,912 patients, PET/CT detected additional FDG-avid primary malignant tumors in at least 1.2% of patients with cancer (2). Recently, dynamic PET/CT (dPET/CT) was developed to obtain absolute quantitative metabolic parameters (e.g., net influx rate Ki, FDG delivery rate K1, and phosphorylation rate k3) by continuously acquiring imaging data over a certain period of time (3). Compared to the standardized uptake value (SUV), a semi-quantitative metabolic parameter of traditional static PET/CT, such as absolute quantitative metabolic parameters have potential advantages in reflecting tumor characteristics and the differential diagnosis of benignity and malignancy (3, 4). Herein, we present a bronchial mass mimicking mediastinal lymph nodes on dynamic ^18^F-FDG PET/CT as a second primary cancer in a 70-year-old woman with a history of gastric carcinoma.

## Case presentation

**Figure 1:**
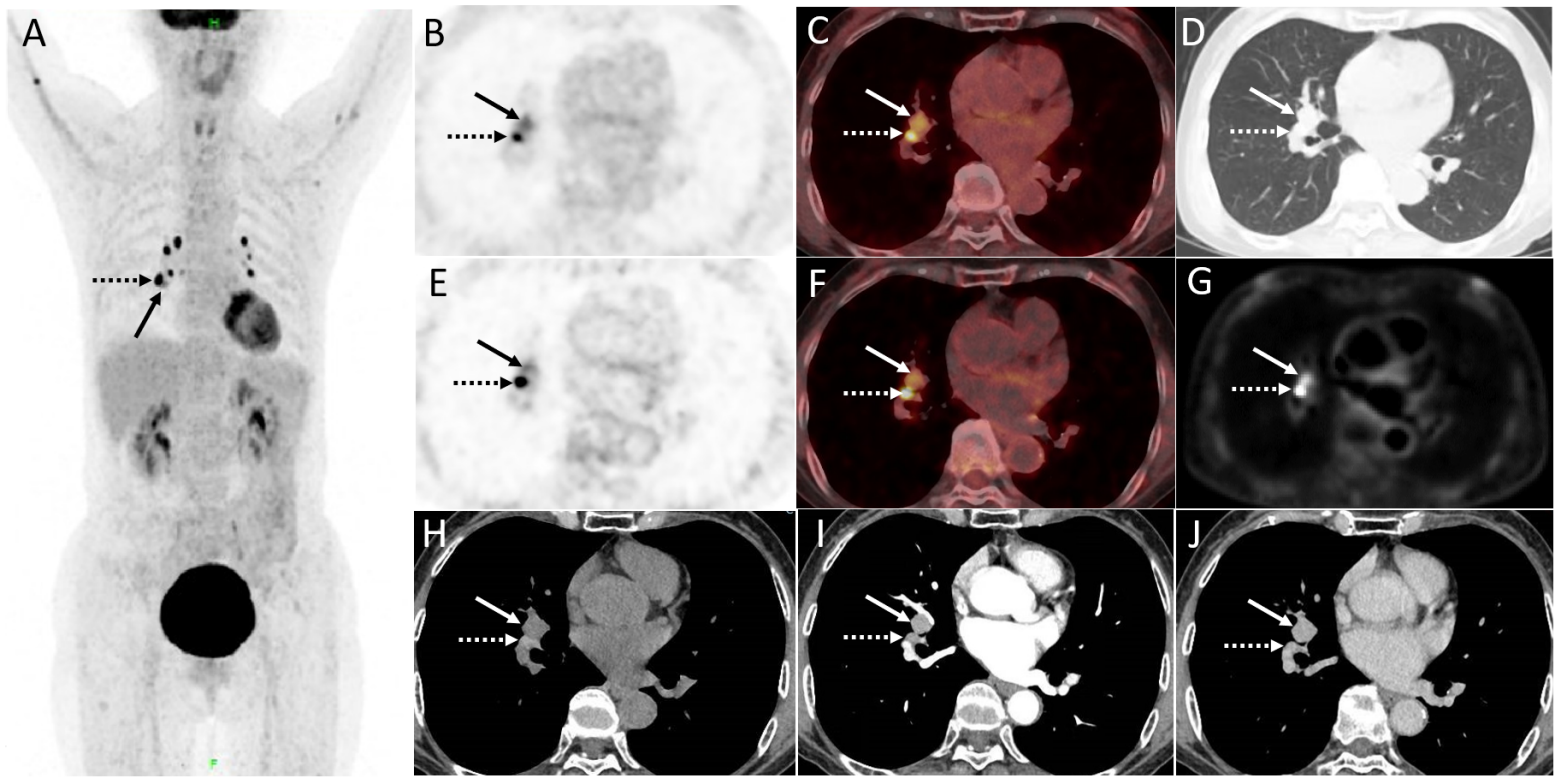
**(A)** The maximum intensity projection image (MIP) of PET/CT. The described right hilar mass (1.6 cm × 1.5 cm) displayed in **(B, C)** regular scan, **(D)** lung window, and **(E, F)** delay scan (arrow, SUVmax of 3.9 and 3.8, respectively) mimicking mediastinal lymph nodes with intense FDG uptake (dotted arrow, SUVmax 12.3 and 17.4. respectively). **(G)** Quantification analysis of dynamic parameters (Ki, net influx rate) shows that the mediastinal lymph nodes had a significantly high uptake of FDG but a relatively low Ki value (average Ki of 0.021 mL/g/min), while the Ki value for endobronchial mass (arrow) was 0.029 mL/g/min. **(H–J)** Contrast-enhanced CT revealed a markedly enhanced endobronchial mass adapting to the right intermediate bronchus.

A 70-year-old woman underwent distal gastrectomy due to gastric adenocarcinoma in 2015. The patient had no complaints of specific discomfort during follow-up, and regular gastroscopy showed no evidence of local recurrence for 6 years after surgery. The blood serum levels of CEA, SCC, NSE, CA125, CYFRA21-1, and ProGRP were normal. Follow-up CT revealed a suspicious mass in the right hilar of the lung mimicking mediastinal lymph nodes. The images of contrast-enhanced CT and dPET/CT are shown in **Figure 1**. The contrast-enhanced CT revealed a markedly enhanced endobronchial mass (1.6 cm × 1.5 cm) adapting to the right intermediate bronchus. Further dPET/CT demonstrated the described right hilar mass with a moderately increased FDG uptake (SUVmax 3.9 and 3.8 for regular and delay scans, respectively) and a relatively high Ki value (0.029 mL/g/min). The symmetrical mediastinal lymph nodes had intense FDG uptake (SUVmax 12.3 and 17.4) but a relatively low Ki value (average Ki of 0.021 mL/g/min).

**Figure 2:**
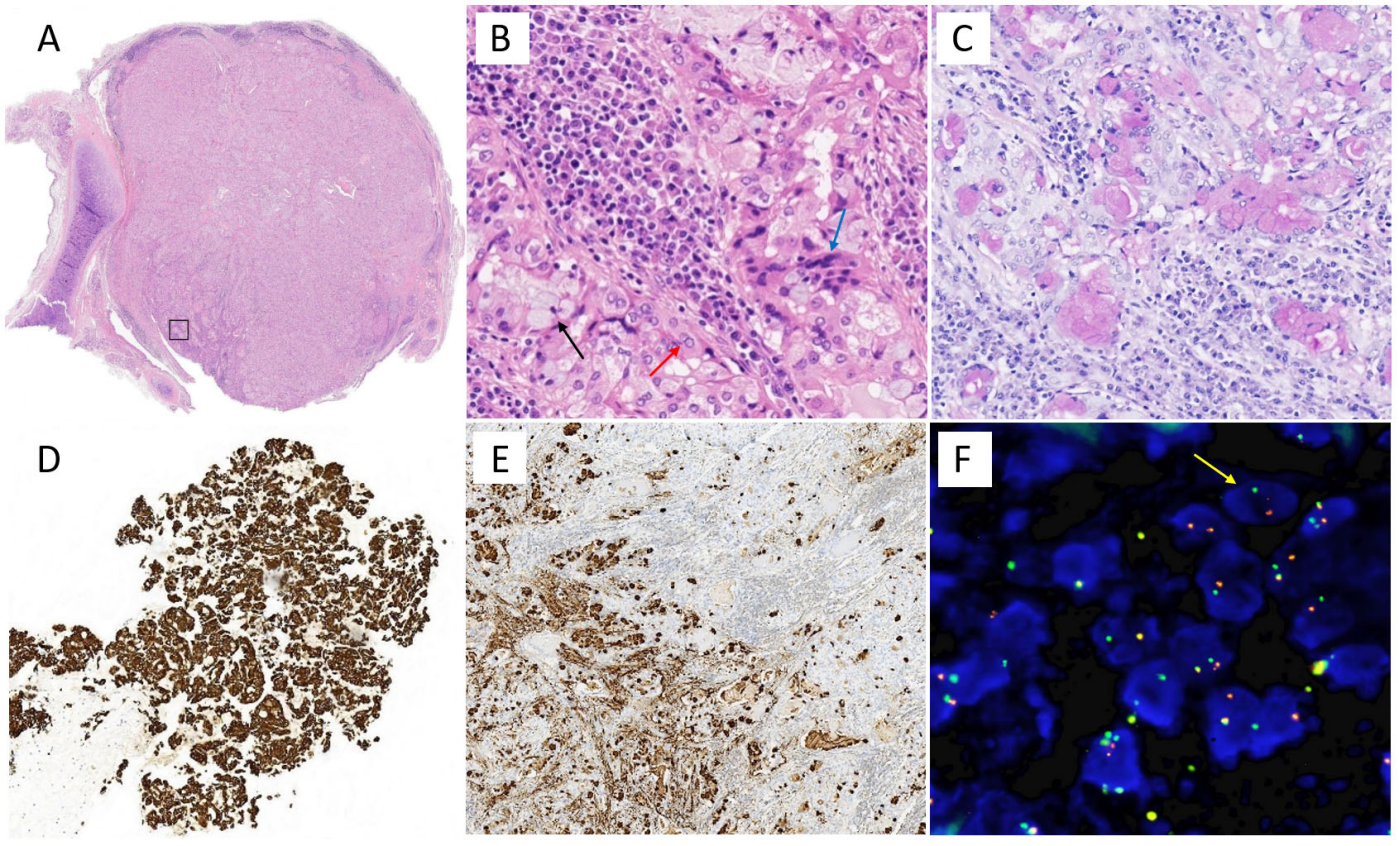
Pathological results of the patient. **(A)** Hematoxylin and eosin stain of ×10 and **(B)** ×400, namely, box area in **(A)**. The tumor was typically a mix of squamous cells (red arrow), mucocytes (black arrow), and intermediate cells (blue arrow). Immunohistochemically, the tumor cells were positive for **(C)** PAS, **(D)** CK7, and **(E)** MUC5AC. **(F)** Dual-color break-apart FISH analysis for the presence of MAML2 gene rearrangement (yellow arrow), which is reported as a diagnostic and prognostic index for PMEC.

The initial pathological result of preoperative bronchoscopy biopsy was considered suspicious for metastatic gastric adenocarcinoma and the EBUS biopsy results of mediastinal lymph nodes in regions 10R and 11L were negative. However, a second primary cancer cannot be ruled out for this single new lesion in an elderly carcinoma survivor, especially given the rarity of tracheal metastasis. Oligometastases or local malignancy may achieve long-term survival after surgery. In addition, the patient was in good condition and had a strong desire for thoracic surgery with the hope of a radical cure. Therefore, the patient then underwent a surgical procedure (lower bilobectomy + hilar and mediastinal lymphadenectomy) for an accurate diagnosis and staging. The pathological result was found consistent with middle-grade mucoepidermoid carcinoma as a second primary cancer without metastasis in 30 resected lymph nodes (**Figure 2**). The tumor was typically a mix of squamous cells, mucocytes, and intermediate cells. Immunohistochemically, the tumor cells were positive for PAS, CK7, and MUC5AC. Dual-color break-apart FISH analysis for the presence of MAML2 gene rearrangement was positive, which is reported as a diagnostic and prognostic index for PMEC (5). Furthermore, the patient was staged as pT1bN0, and no further treatment was deemed necessary. The regular follow-up during 2.5 years after surgery showed no signs of abnormality.

## Discussion

For a patient with a previous cancer history, it can be difficult to establish the diagnosis of an additional primary, which is not uncommon nowadays. An unexpected bronchial mass found in a patient with known cancer was likely to be mistaken as mediastinal lymph nodes or tracheobronchial metastasis, which is rarely seen. Wumener et al. reported the value of dPET/CT in differentiating malignant and benign lymph nodes (6) and lung lesions (4) with the cutoff value of Ki as 0.022 mL/g/min and 0.025 mL/g/min, respectively. Despite the significant high uptake of FDG of mediastinal lymph nodes in this case, the bilateral symmetrical distribution and relatively low Ki value suggest benign lesions consistent with pathological results. Whereas the bronchial mass with moderately increased FDG uptake but a relatively high Ki value suggests its malignancy. On the other hand, tracheobronchial metastasis is rare and occasionally reported in cases with melanoma, breast, colorectal, and renal cancers (7).

Moreover, a single new lesion several years after a primary cancer diagnosis should alert clinicians to the possibility of a second primary tumor, especially in older adults (1). Approximately one-third of cancer survivors aged > 60 years have been diagnosed more than once with another cancer (8). Pulmonary mucoepidermoid carcinoma (PMEC) is extremely rare, with an estimated incidence of less than 1% of all primary lung neoplasms, and the majority of PMEC occurs in adults between 30 and 40 years of age (9). PMEC is classified as a salivary gland-type tumor originating from the submucosal glands of the tracheobronchial tree, which is difficult to diagnose by limited biopsy via bronchoscope (10). PMEC usually appears as oval or lobulated proximal airway masses with an occasionally punctate calcification (11), and shows different extents of FDG uptake, varying from 0 to 10.7 for low-grade and 2.9 to 23.4 for high-grade (10, 12–15), with a reported optimal cutoff of 6.5 (16). Several studies have reported that PMEC can present as multiple primary lung cancer (MPLC) with squamous or small cell lung cancer (17), or mixed lung cancer comprising mucoepidermoid carcinoma and conventional adenocarcinoma (18). However, to our knowledge, pulmonary mucoepidermoid carcinoma as a second primary cancer with a different origin from other tissues and organs has not been reported.

In conclusion, ^18^F-FDG PET/CT has an important value in the follow-up of indeterminate findings for patients with a tumor history. Moreover, dynamic quantification parameters such as Ki may be additionally helpful in identifying malignancies in some equivocal situations.

## Data Availability

The original contributions presented in the study are included in the article/supplementary material. Further inquiries can be directed to the corresponding author.
